# Improving translatability of preclinical studies for neuromuscular disorders: lessons from the TREAT-NMD Advisory Committee for Therapeutics (TACT)

**DOI:** 10.1242/dmm.042903

**Published:** 2020-02-07

**Authors:** Raffaella Willmann, Joanne Lee, Cathy Turner, Kanneboyina Nagaraju, Annemieke Aartsma-Rus, Dominic J. Wells, Kathryn R. Wagner, Cristina Csimma, Volker Straub, Miranda D. Grounds, Annamaria De Luca

**Affiliations:** 1 Swiss Foundation for Research on Muscle Diseases, 2016 Cortaillod, Switzerland; 2 John Walton Muscular Dystrophy Research Centre, Centre for Life, Newcastle University and Newcastle Hospitals NHS Foundation Trust, Newcastle upon Tyne NE7 7DN, UK; 3 School of Pharmacy and Pharmaceutical Sciences, Binghamton University, New York, NY 13902-6000, USA; 4 Department of Human Genetics, Leiden University Medical Center, Leiden, 2300 RC, the Netherlands; 5 Neuromuscular Disease Group, Royal Veterinary College, London NW1 0TU, UK; 6 Center for Genetic Muscle Disorders, Kennedy Krieger Institute and the Departments of Neurology and Neuroscience, Johns Hopkins School of Medicine, Baltimore, MD 21205, USA; 7 Csimma LLC, Lincoln, MA 01773, USA; 8 School of Human Sciences, The University of Western Australia, Perth, WA 6009, Australia; 9 Unit of Pharmacology, Department of Pharmacy and Drug Sciences, University of Bari Aldo Moro, 70125 Bari, Italy

**Keywords:** Animal models, Preclinical, Guidelines, Efficacy studies, Clinical trial, Standard protocols, Neuromuscular

## Abstract

Clinical trials for rare neuromuscular diseases imply, among other investments, a high emotional burden for the whole disease community. Translation of data from preclinical studies to justify any clinical trial must be carefully pondered in order to minimize the risk of clinical trial withdrawal or failure. A rigorous distinction between proof-of-concept and preclinical efficacy studies using animal models is key to support the rationale of a clinical trial involving patients. This Review evaluates the experience accumulated by the TREAT-NMD Advisory Committee for Therapeutics, which provides detailed constructive feedback on clinical proposals for neuromuscular diseases submitted by researchers in both academia and industry, and emphasizes that a timely critical review of preclinical efficacy data from animal models, including biomarkers for specific diseases, combined with adherence to existing guidelines and standard protocols, can significantly help to de-risk clinical programs and prevent disappointments and costly engagement.

## Abstract (precis)

**Summary:** Rigorous preclinical efficacy studies de-risk clinical trials in neuromuscular diseases. This Review summarizes the impact of expert panel advice on translational studies.

## Introduction

Animal models of human genetic diseases are useful for several reasons. Firstly, they allow the study of mechanisms of a specific disease at different stages and in association with development, growth and aging, by sampling different types of tissues and fluids, and by assessing the animals functionally in a manner that is relevant to the disease. This facilitates a better understanding of the disease and aids in identifying potential new therapeutic targets. Secondly, animal models enable the testing of experimental therapies. For neuromuscular diseases, much of the knowledge we have today about how muscle conditions evolve, and which pathways are affected, derives from studies in mice and other model organisms. This vital *in vivo* knowledge helps to identify druggable targets and to design or test specific therapeutic modalities, as well as to detect diagnostic or therapeutic biomarkers. Animal models also allow the comparison of pathological phenotypes with the human disease, and focused studies may help to gain insight into the underlying reasons for differences at varying levels of complexity. This can also have an important impact on the translational effort, allowing the identification of protective pathways in animals that could be enhanced in humans.

Animal models are widely used to preclinically test novel therapeutics, including small molecules, biologics, gene modifiers and cell therapies. However, translation of a potential therapy with proven preclinical efficacy to the human setting is much more challenging. In part, this is owing to differences in the nature of the disease pathology in humans. Some inherited neuromuscular diseases have the advantage of a clear genotype-phenotype correlation allowing more direct approaches via the modulation of the main target of the pathology (e.g. certain muscle channelopathies). Others, although monogenic, are more complex, as a mutation may not be fully penetrant, or the monogenic defect can trigger a complex cascade of pathological events and the mutant allele may exhibit variations that depend on the broader genetic environment. Also, certain mouse genetic backgrounds may include genetic modifiers that influence the overall manifestation of pathology and make it harder to translate drug efficacy to humans. However, even these so-called ‘imperfect’ models offer a unique opportunity to gather essential information on the pathogenesis and on the targeting of specific pathways for therapy development, provided one is aware of the limitations of the model and takes them into consideration when interpreting the data and translating it to the clinical setting.

One important, and often underestimated, aspect to consider when evaluating clinical translatability is the rigor and robustness of the preclinical studies and their reproducibility, which allows comparison and confirmation of results from different labs. In many instances, a candidate therapy proceeds to a clinical setting, especially for a limited patient population, based on tissue culture studies or an ‘extraordinary’ proof-of-concept finding in an animal model without wider preclinical validation, paving the way for a highly risky and most likely suboptimal process of translation to the clinic. In fact, this process frequently fails owing to an underestimation of crucial variables. Such clinical failure is often explained by an intrinsic limitation of the animal model in fully recapitulating the human disease. But in reality, it may be due to a lack of strong preclinical data for the proposed therapy, including optimal dosing regime and route. Overall, this triggers a loss of faith in the usefulness of model animals for the development of treatments. Although the quick translation of therapeutic compounds into clinical trials, based on premature preclinical results, is commonly used to move forward in the drug development process (Kimmelman et al., 2014), it in fact hampers it by increasing the chance of failure, where it is unclear whether the clinical trial failed because the test compound was ineffective or because the trial rationale or design were suboptimal (Scott et al., 2008). In the context of rare diseases, this is aggravated by the participation of the already very small number of patients in trials that have limited potential to succeed and/or provide a clear outcome.

### Background

Launched in 2007, the EU-funded network of excellence for genetic neuromuscular diseases, Translational Research in Europe for the Assessment and Treatment of Neuromuscular Disorders (TREAT-NMD; FP6 contract number EC 036825), aimed at addressing the clinical translation problem by harmonizing best practices and tools in Europe to accelerate the development of effective treatments for neuromuscular diseases, with an initial focus on Duchenne muscular dystrophy (DMD) and spinal muscular atrophy (SMA). The network quickly expanded outside European boundaries and embraced other neuromuscular diseases, for which standards of care, international registries and biobanks were developed and launched. In the preclinical research space, it was clear that the fragmentation of research approaches and the lack of guidelines to define the level of rigor required to promote a preclinical study for translation into humans were key aspects to address. TREAT-NMD strongly promoted an ambitious international effort to develop Standard Operating Procedures (SOPs) under the consensus of key scientists for the assessment of the most important animal readouts in a more reproducible way (Grounds et al., 2008; Nagaraju and Willmann, 2009; Willmann et al., 2011; see also https://treat-nmd.org/research-overview/preclinical-research/). In place since 2009, the use of SOPs is increasingly required by the National Institutes of Health and other funding bodies for grant assessment or also in preparation of scientific advice meetings on investigational new drugs. Accordingly, the use of SOPs is mentioned in several publications (for example George Carlson et al., 2011; Israeli et al., 2019; Mantuano et al., 2018; Mele et al., 2019; Tam et al., 2015; Zschüntzsch et al., 2016). The TREAT-NMD website has recorded more than 11,000 SOP downloads over 7 years. The network progressively developed additional guidelines and recommendations, and held workshops to improve the quality and robustness of preclinical studies, to increase transparency in reporting and as independent validation (Willmann et al., 2015, 2012). This effort with multiple stakeholders is highly dynamic, continuously involving novel animal models and technical advancement in experimental strategies (see for example Gordish-Dressman et al., 2018; Willmann et al., 2018).

TREAT-NMD also strived to consolidate a ‘modus operandi’ that could be widely accepted by the community for the sake of the community itself: all interested stakeholders, including editors and funding agencies, should be aware that the quality of preclinical data for translational research needs to be predominantly robust, reproducible and less focused on novel and sensational results (Willmann et al., 2018). This process can be in part ensured with a clear distinction between proof-of-concept preclinical studies, often aimed at target validation with an interesting candidate molecule, and clinically-oriented ones that are aimed at the translational effort and that are built with the rigor that resembles clinical trials and/or the toxicology data package required for drug approval (Grounds et al., 2008; Willmann et al., 2015).

### The TREAT-NMD Advisory Committee for Therapeutics

The TREAT-NMD Advisory Committee for Therapeutics (TACT, launched in 2009) is a unique multi-disciplinary international group of well recognized neuromuscular healthcare, academic and industry drug development experts, as well as representatives of patient organizations and experts in regulatory affairs. The aim of TACT is to review therapy development programs submitted by academia or industry and provide guidance and advice on the different aspects of translational research. An overall view of TACT's structure and outputs after 5 and 10 years of activity has been described in Heslop et al. (2015) and Wagner et al. (2019). This Review focuses on the challenges of the preclinical phase of drug development and aims to recommend measures to avoid the waste of resources, making use of the experience collected over the 10 years of the TACT program.

A schematic of the typical TACT proposal workflow is available on https://treat-nmd.org/tact-treat-nmd-advisory-committee-for-therapeutics/tact-application-process/. Whatever the stage of the program application submitted to TACT, the committee performs a careful evaluation of preclinical data in order to provide guidance or to uncover possible bias that could negatively affect the clinical trial design, or on the proper identification of therapeutic biomarkers and clinically meaningful endpoints. The robustness of the available data is checked according to the guidelines developed by TREAT-NMD and the SOPs available or, alternatively, according to the wider scientific community's best knowledge available on the animal model or preclinical setting proposed in the program application.

Based on the experience accumulated by TACT, we analyze here the compliance with guidelines and standards in the neuromuscular research community, evaluate the effort to improve translational efficacy for the benefit of patients, and estimate the impact that TACT has had on the development and success of clinical trials for neuromuscular diseases.

## Results

### TACT advice on the preclinical assessment of therapeutic programs from 2010-2019

In 10 years of activity up to April 2019, TACT has provided guidance to 56 development programs. At the time of writing this Review, 59% were in the preclinical phase, 32% were in clinical trial phase I and 9% were in phase II.

During the evaluations of the preclinical programs submitted to TACT, some common themes emerged from the review reports (Table 1). Inappropriate doses/administration route, inappropriate readouts or inappropriate tissue choice for a given readout were the most frequent issues identified by TACT. Randomization, blinding and appropriate controls were missing in 30% of the programs submitted to TACT (Table 1). The availability and the proper use of biomarkers in the preclinical phase depends on the disease and on the expected mechanism of action of the drug. For the general purpose of this Review, we therefore decided not to include this issue in the overall evaluation of the preclinical programs.

**Table 1.**
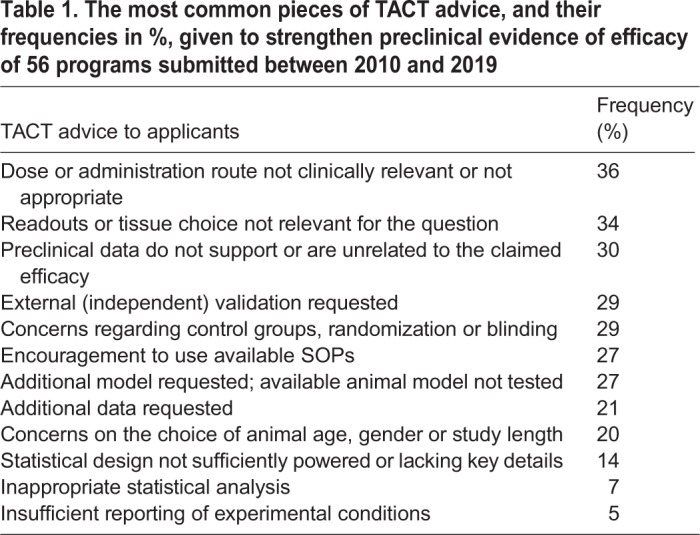
**The most common pieces of TACT advice, and their frequencies in %, given to strengthen preclinical evidence of efficacy of 56 programs submitted between 2010 and 2019**

**Fig. 1.**
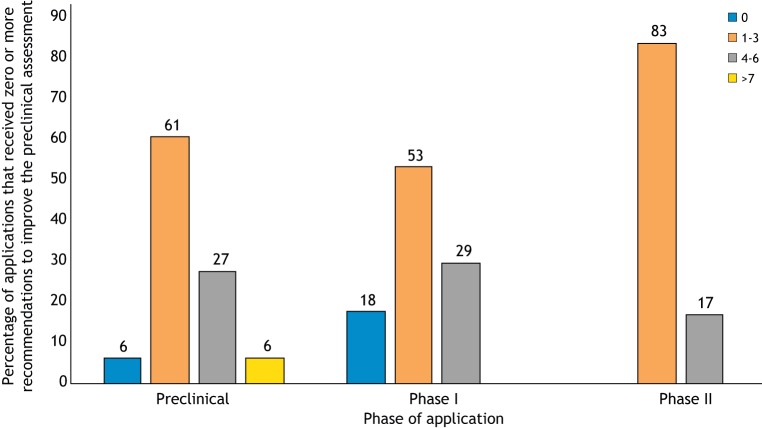
**Recommendations on preclinical data given to TACT applicants.** The *x*-axis shows the number of items of TACT advice (see Table 1) given to applicants in relation to the development phase of the research program at the application date. For example, 61% of programs submitted at the preclinical phase were given between one and three items of TACT advice.

In general, only few programs had no issues on the preclinical assessment (9%). Most programs were given one to three of the advices listed in Table 1. Almost 30% of the programs submitted to TACT at preclinical stage or at phase I clinical trial stage, and 17% of the programs submitted at phase II clinical trial stage, were given four to six pieces of advice to strengthen the preclinical assessment (Fig. 1).

**Fig. 2.**
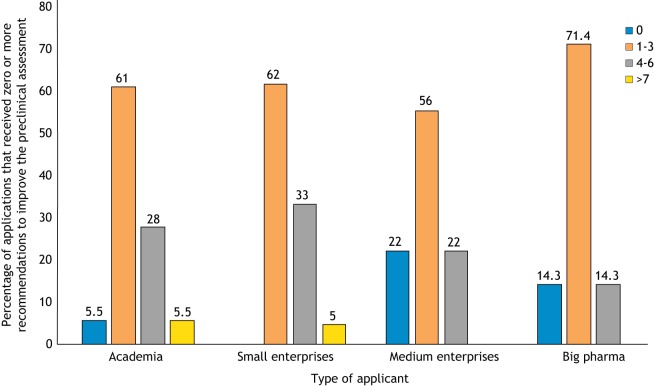
**Distribution of TACT advice according to applicant type.** The *x*-axis shows the percentage of programs for which TACT identified pre-clinical issues. For example, in 61% of the programs coming from academia, TACT provided one to three items of advice in the preclinical assessments.

Applications originated from academia (32%), from small companies with 50 employees or fewer (45%), from medium-sized companies with 51-250 employees (7%) and from big pharma companies with more than 250 employees (16%) (Wagner et al., 2019). In relation to the type of applicant, most programs that did not receive any additional recommendation for their preclinical assessment were submitted by medium-size enterprises and big pharma. However, all categories of applicants submitted programs that did receive recommendations on their preclinical development (Fig. 2).

### Development of clinical trials after TACT advice

**Fig. 3.**
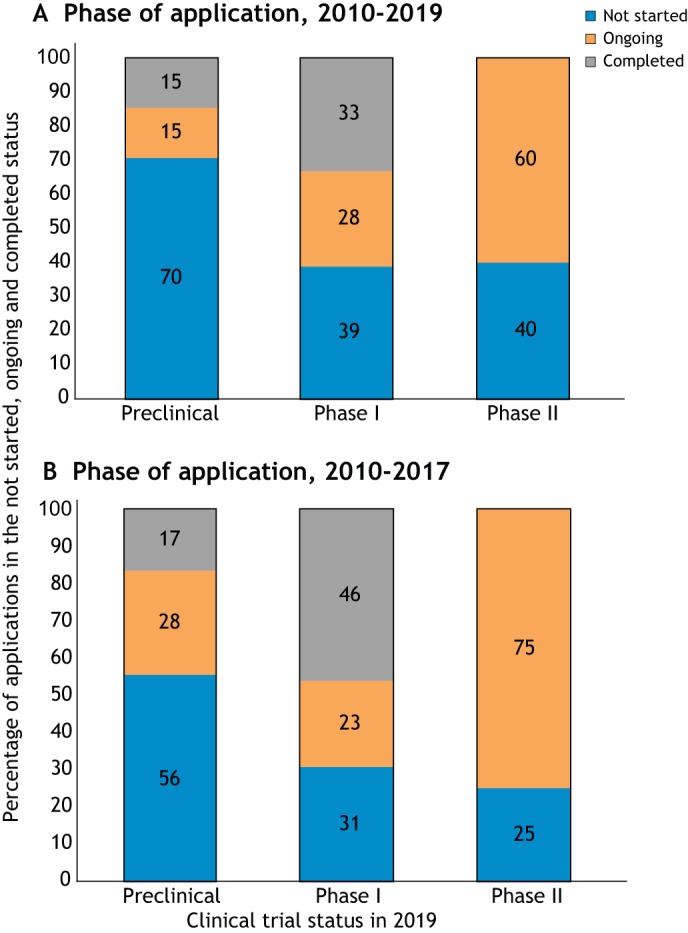
**Clinical trial status in 2019 related to stage of development at the TACT submission date.** (A) Clinical trial status in 2019 related to stage of development at the TACT submission date 2010-2019. Programs that were submitted at their preclinical phase have not yet resulted in a clinical trial in 70% of the cases (blue bar), whereas 30% (orange and gray bars) resulted in trials that are still ongoing or completed. (B) Clinical trial status in 2019 related to stage of development at the TACT submission date 2010-2017. Considering programs submitted until spring 2017, the rate of continuing into clinical trials slightly increases.

We reviewed the number of TACT programs that progressed to clinical trials over the years. Fewer programs that were reviewed by TACT at a preclinical stage progressed to a subsequent clinical trial (30%) than programs reviewed at a later planning phase (60%, Fig. 3A).

Considering that the initiation of a clinical trial is a lengthy process, we redrew this figure considering only programs submitted until spring 2017 and observed a slight increase in these percentages: 45% of programs submitted at preclinical stage and 69-75% of programs submitted at a later stage result in an ongoing or completed trial in 2019 (Fig. 3B).

**Fig. 4.**
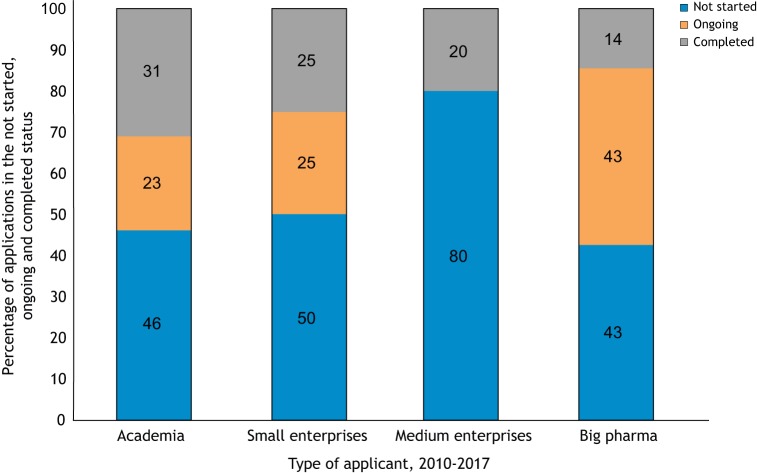
**Clinical trial status in 2019 related to applicant type, 2010-2017.** Programs submitted before spring 2017 developed into a clinical trial in ∼50% of the cases (orange and gray bars).

However, the rates of progress to clinical trials (ongoing and completed trials) is similar for all categories of applicant, with a clearly lower success rate observed for medium enterprises (Fig. 4). This could be explained by the fact that of the five medium-sized enterprises that applied for a TACT review up to 2017, two did not have a focus on neuromuscular diseases at the date of application. Data in Fig. 4 suggest that the higher number of programs submitted to TACT at a preclinical stage and not yet progressed to clinical trials, as shown in Fig. 3A, probably reflects the value of early-stage feedback, which presents the opportunity to revise the trial design and planning. TACT advice may have indicated the importance of additional preparatory work asking the right questions early in program development. An objective critical assessment in the early stage of planning of a clinical program is essential to avoid the waste of resources that can result from premature initiation of a suboptimal clinical trial.

**Fig. 5.**
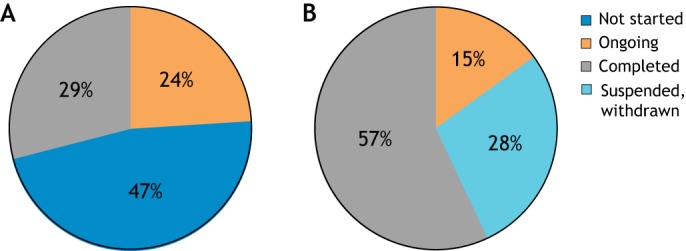
**Status of clinical trials in 2019 for DMD programs.** (A) Status of clinical trial in 2019 for DMD programs submitted to TACT 2010-2017. (B) Status of all DMD clinical trials in 2019 registered on clinicaltrials.gov 2010-2017. Almost 30% of the DMD trials needed to be stopped ahead of completion, indicating a high rate of failure that could probably be reduced by a careful evaluation of the preclinical evidence and its readiness for translation to the clinical setting.

Indeed, this can be partly confirmed by considering the percentage of trials registered on the clinicaltrials.gov portal that needed to be withdrawn. To simplify, we looked at DMD trials only, based on the fact that the great majority of TACT-reviewed programs developed interventions for DMD (Heslop et al., 2015). We compared the number of trials for DMD that underwent a TACT review but were never started with the global number of trials for DMD that were registered on clinicaltrials.gov in the same time period but were stopped before conclusion. Fig. 5A shows that, in this subset, almost half of the trials were never started after their proponents received TACT advice. In the same time period, 30% of interventional trials in DMD registered on clinicaltrials.gov had to be withdrawn or terminated ahead of time (Fig. 5B). This supports the observation that a timely critical review of the preclinical data may prevent the translation of a weak program into a patient trial, and may result in the reduction of unsuccessful clinical trials. Indeed, only one of the 37 TACT-reviewed programs on DMD that eventually progressed to a clinical trial was discontinued, whereas 36 are still ongoing or are completed.

## Discussion

In the field of rare diseases, researchers need to plan clinical trials particularly well. This is because, even in the best scenarios, the numbers of eligible patients are limited. It is further complicated by the fact that most rare diseases have no therapies available, and therefore often lack post-marketing, real-world data. Owing to the unmet medical need, unsuccessful or terminated clinical trials are generally very damaging for the communities involved. Researchers increasingly recognize that most preclinical experiments do not represent a true preclinical efficacy study. In the past decades, discussions on the value of mouse models in predicting a therapy's efficacy for humans concluded that preclinical efficacy studies for therapies that aim at transition to clinical trials should be conducted with the same rigor as the clinical trials themselves (Conwit et al., 2011; Knopp et al., 2015). To help achieve high-quality preclinical studies, primary and secondary outcomes should be defined in advance and assessed according to standardized protocols, adequate control groups need to be chosen, and power analysis should be used to determine sample size; randomization and blinding need to be implemented, and a careful statistical analysis should be applied. The choice of readouts and biomarkers needs to be tailored to the drug tested and to the expected efficacy, both on the molecular level and for functional, clinically relevant aspects. Notably, as in human trials, preclinical studies can only be properly planned when the natural history of the model system is known (see for example Gordish-Dressman et al., 2018; van Putten et al., 2019; Verhaart et al., 2019), as this will also guide researchers in determining when to start the intervention and when and how to assess its therapeutic effects.

However, there are no predetermined rules, for example by regulatory authorities, to define the conduct of clinically-oriented preclinical studies for rare diseases, and the enthusiasm about new findings sometimes leads to haste and unrealistic expectations of translation to humans. This frequently means a lack of rigorous controls, independent validation and proper sample sizes. Aware of this problem, TACT carefully reviews all the preclinical data in the submitted programs. Where data are missing or unclear, the committee explicitly recommends clarification or additional items. A recent analysis of the adequacy of the mouse versus dog model for DMD, according to the question posed, confirms that researchers need to make a careful choice before assessing the efficacy of treatments in animals (Ferreira et al., 2019).

Despite the broad discussion on the need for rigor in preclinical studies and the publication of recommendations, guidelines and consensus papers on this topic (Bøtker et al., 2018; Henderson et al., 2013; Smith and Houghton, 2013; Tuzun et al., 2015), basic issues are still present in much of the preclinical efficacy data submitted to TACT. These include a lack of randomization, blinding and adequate controls (Table 1). In the 10 years of TACT experience, it has become clear how often preclinical data, which are used as the rationale to start a human trial, lack one or more of the basic recommendations. The percentage of programs submitted to TACT that attempted the transition to clinical trials based on poor preclinical efficacy studies predicts the risk of unsuccessful trials (Fig. 1). Both academia and industry planning clinical trials submitted programs containing preclinical studies that were missing up to eight of the 12 basic aspects regarded as key for meaningful preclinical efficacy studies, with the highest numbers occurring in programs submitted by academia and small- to medium-sized enterprises (Fig. 2).

It is essential to underscore again, at this stage, that exploratory and proof-of-concept studies are different from the subsequent preclinical (translationally-oriented) efficacy ones. The former raise treatment hypotheses or use drugs to validate a pathology-related pathway and can generate much excitement. The latter typically follow proof-of-concept work and are carefully designed with the aim to test and prove the efficacy for a possible future clinical application on humans. The existing guidelines and their required rigor are necessary for the latter category of preclinical studies. Specific SOPs for the assessment of readouts in distinct animal models now provide researchers with a platform of standardized methods for proper use of animal models, in order to obtain robust and reproducible results. According to this point, independent validation of a preclinically tested compound is another key point to consider (Capogrosso et al., 2018).

We observed that a critical assessment of the preclinical data meant to support a future clinical trial in rare diseases (as assisted by TACT) may contribute to more time invested in robust conclusive preclinical studies to strengthen efficacy data and reduce the risk of unsuccessful clinical trials (Figs 3–5). At the same time, this also avoids including patients in trials that have a low probability of success, and the associated exclusion of the same limited pool of patients from more promising trials.

## Conclusions

In spite of the 15 years of efforts in educating and raising awareness within the academic and industry communities about the importance of rigor in clinical translation-oriented animal studies for rare diseases, this ‘real world’ analysis through TACT reviews underlines that the quality of preclinical data supporting the ‘efficacy’ of a drug or therapy is too often still limited or scattered. Although it is accepted that animal studies are necessary to provide data on the *in vivo* mechanism, target engagement, efficacy, dosage and biomarker development, the need for more preclinical studies to enhance data quality is questioned based on the often-quoted statement that animal models poorly represent human disease. Actually, an animal model, when properly used, can be highly informative and prevent the huge waste of resources, time, and imposition on patient populations of poorly justified or poorly designed clinical trials. Validation of target engagement in proof-of-concept studies and assessment of efficacy in properly designed clinically-oriented preclinical studies can also support toxicology studies, and help dose finding and pharmacokinetic/pharmacodynamic assessments (Heier et al., 2013; Meyer et al., 2015).

Although the requirements for toxicology assessment of new drugs are clearly defined, no regulation is available for the preclinical pharmacology data used to support a human trial (Langhof et al., 2018). Similarly, the absence of requirements for reporting standards in scientific publications has led to efforts in compiling ARRIVE guidelines (Kilkenny et al., 2010), but their implementation by journals is still very poor (Hair et al., 2019). Therefore, the scientific, clinical and industry communities, in parallel with regulators, need to be actively aware of the risk of using a poor data set from animal models for premature translation to a human trial.

Based on the observations discussed in this Review, TACT suggests referring to the TREAT-NMD guidelines and SOPs as a checklist to plan clinically-oriented preclinical studies and improve the strength of the scientific rationale for a clinical trial. Although in 2015, 72% of all programs submitted to TACT developed interventions for DMD (Heslop et al., 2015), in 2019 this was reduced to 66%, and TACT registered an increased broader development of treatments for other diseases (Wagner et al., 2019). Therefore, a standard evaluation of existing animal models for less common neuromuscular diseases and the generation of specific SOPs and guidelines would help the wider research community conducting reproducible and robust preclinical efficacy studies.

At the moment, there are not enough ongoing trials that originally applied for TACT advice to draw conclusions about the impact of the advisory feedback on successful development of a therapeutic. However, the collected experience suggests that TACT may better help applicants whose translational projects are in the early phases of development, provided that a candidate drug at good manufacturing practice quality and ready for human use, including its main toxicology data set, is available. Concluding from the remarks of many TACT applicants (see Box 1, Feedback from past TACT applicants), having multidisciplinary drug development experts at the table is considered valuable and important to independently review the scientific rationale for a drug. Definitively, an appropriate robust preclinical data package has a chance to de-risk, prevent disappointments, reduce costs and avoid unnecessary and highly taxing engagement of a limited patient population.

**Box 1. Feedback from past TACT applicants**

‘We have rethought our list of priorities. We are now focusing on efficacy in mouse and have stopped experiments in other models. We have started blinded experiments with different doses, and we have defined primary and secondary outcomes to measure efficacy.’

Ainara Vallejo, Biodonostia Research Institute

‘This is a great way to pressure test thinking, hear additional perspectives on our program and “practice” for meetings with the EMA and FDA.’

Suyash Prasad, Audentes Therapeutics

‘The TACT committee had an outstanding variety of expertise that was extremely useful to cover all topics and issues of our program.’

Ramon Martí, Vall d'Hebron Research Institute

‘This was the best way to examine all aspects of the program at once and make sure everything fitted together. I would request a TACT advice process again.’

Deborah Ramsdell, Valerion Therapeutics

This article is part of a special collection ‘A Guide to Using Neuromuscular Disease Models for Basic and Preclinical Studies’, which was launched in a dedicated issue guest edited by Annemieke Aartsma-Rus, Maaike van Putten and James Dowling. See related articles in this collection at http://dmm.biologists.org/collection/neuromuscular.
